# Vigorous physical activity is associated with mental well-being: indirect associations via resilience and physical self-esteem in university students

**DOI:** 10.3389/fpsyg.2025.1747633

**Published:** 2026-01-23

**Authors:** Manman Shi, Xia (Sebastiane) Chen, Binfeng Yuan

**Affiliations:** 1 Guangdong University of Finance & Economics, Guangzhou, China; 2 University of Wisconsin – Milwaukee, Milwaukee, WI, United States

**Keywords:** mental well-being, physical self-esteem, resilience, serial mediation, vigorous physical activity

## Abstract

This cross-sectional study examined whether resilience and physical self-esteem statistically account for the association between vigorous physical activity (VPA) and mental well-being among university students. A total of 2,432 undergraduates from three universities in Guangdong, China completed validated measures of VPA (IPAQ-SF), resilience (CD-RISC-10), physical self-esteem (PSPP–Physical Self-Worth), and mental well-being (WHO-5). After confirming the measurement model, a hypothesized serial mediation model (VPA → resilience → physical self-esteem → mental well-being) was tested using structural equation modeling. The model demonstrated acceptable fit (CFI = 0.952, TLI = 0.944, RMSEA = 0.047, SRMR = 0.039). VPA showed positive associations with resilience (*β* = 0.24, *p* < 0.001) and physical self-esteem (*β* = 0.20, *p* < 0.001). Resilience was positively associated with physical self-esteem (*β* = 0.38, *p* < 0.001) and mental well-being (*β* = 0.36, *p* < 0.001), and physical self-esteem was positively associated with mental well-being (*β* = 0.24, *p* < 0.001). The direct association between VPA and mental well-being remained small but significant (*β* = 0.06, *p* < 0.01). Bias-corrected bootstrapping (5,000 resamples) indicated significant indirect associations via resilience, via physical self-esteem, and through the serial pathway, although the serial indirect effect was small in magnitude. These findings are consistent with partial statistical mediation and suggest that interventions may benefit from integrating vigorous physical activity with resilience-building and competence-enhancing components. Given the cross-sectional design, the results reflect associations and do not permit causal inference. Mental well-being was operationalized as positive well-being, with higher WHO-5 scores indicating better well-being.

## 1 Introduction

University students experience substantial mental health challenges, making modifiable health behaviors a focus for prevention on campuses. Recent syntheses and large student samples indicate that greater physical activity is associated with fewer symptoms of depression, anxiety and psychological distress in undergraduates, and that activity intensity may matter (Huang et al., 2024, 2025). In addition, population guidance emphasizes the benefits of vigorous physical activity (VPA)—typically ≥75–150 min/week—supporting our decision to examine vigorous activity specifically in a university cohort (World Health Organization, 2020). Evidence from Chinese university students further suggests that low MVPA combined with high screen time confers the highest odds of psychological symptoms, underscoring the relevance of activity dose in this age group (Deng et al., 2024).

Mechanistic research increasingly points to psychological resilience and physical self-esteem/self-perception as key mediators of the activity–mental well-being link in youths and university students. A 2024 best-evidence review concluded there is strong support for physical self-worth and growing support for other psychosocial mediators between physical activity and mental well-being (White et al., 2024). Meta-analytic evidence shows a positive association between physical activity and resilience in young students (Qiu et al., 2025), and university-based studies report that physical self-perception or physical self-esteem mediates associations between physical activity and well-being/quality of life (Chen et al., 2025; Zhu et al., 2025). Related student studies also find self-esteem mediates physical activity’s effects on other psychosocial outcomes (Ke et al., 2024).

However, few studies have integrated these mechanisms into a single serial pathway in which VPA is associated with mental well-being, with indirect associations consistent with resilience (M1) and subsequently physical self-esteem (M2) among university students. Existing chain-mediation models in this population often involve different mediator combinations—e.g., resilience with sleep (Li et al., 2024) or mindfulness with resilience (Guo, 2025)—highlighting a gap our model addresses. Accordingly, we test a serial mediation model—VPA → resilience → physical self-esteem → mental well-being—and hypothesize a positive total association of VPA with mental well-being plus significant indirect associations through resilience and physical self-esteem, including a serial indirect association.

## 2 Literature review and research hypothesis

### 2.1 Vigorous physical activity and student mental well-being

Across university populations, higher physical activity relates to fewer symptoms of depression, anxiety, and psychological distress, with several studies highlighting dose/intensity effects (Du et al., 2025). In Chinese undergraduates, low MVPA together with high screen time is linked to the greatest odds of psychological symptoms, and fresh evidence suggests that vigorous-intensity activity shows stronger associations with lower depression and anxiety than moderate activity (Du et al., 2025). Public health guidance likewise emphasizes benefits of accumulating at least 75–150 min/week of vigorous activity for adults (World Health Organization, 2020). Collectively, these findings justify focusing on VPA as the exposure of interest in this study. (H1 below).

*H1.* Vigorous physical activity is positively associated with mental well-being among university students.

### 2.2 Psychological resilience as a mediator

Mechanistic work increasingly implicates psychological resilience—adaptive capacity under stress—as a pathway linking activity to mental well-being. A 2025 meta-analysis of young students (including college samples) reported a significant, moderate positive association between physical activity and resilience (Qiu et al., 2025). In undergraduates, resilience frequently functions as a mediator or chain mediator connecting exercise to better mental well-being, and has been shown to partially account for links between exercise and reduced “mental sub-health” (Zhang et al., 2025). These findings support positioning resilience as M1 in our model. (H2 below).

*H2. Psychological resilience statistically mediates the association between vigorous physical activity and mental well-being.*

### 2.3 Physical self-esteem and an integrated serial pathway

The Exercise and Self-Esteem Model (EXSEM) and contemporary extensions posit that exercise enhances physical self-perceptions (e.g., physical competence/appearance), which in turn elevate global self-esteem and downstream well-being (Wong et al., 2021). In university samples, physical self-perception/physical self-esteem mediates associations between physical activity and quality of life or well-being. Importantly, resilience is also linked to competence-related beliefs in exercise contexts: recent work shows resilience mediates the effect of physical activity on physical self-efficacy (a construct adjacent to physical self-esteem within hierarchical self-models), suggesting a plausible serial pathway from VPA → resilience → body-related self-views → mental well-being; see also (White et al., 2024), for mediator evidence across the PA–mental well-being literature. We therefore test an integrated chain. (H3–H4 below).

*H3.* Physical self-esteem statistically mediates the association between vigorous physical activity and mental well-being.

*H4.* Vigorous physical activity shows an indirect serial association on mental well-being via resilience (M1) and then physical self-esteem (M2).

## 3 Methods

### 3.1 Participants

Undergraduate students were recruited from three universities in Guangdong Province, China—Guangdong University of Finance and Economics, Jinan University, and Guangdong University of Technology—to complete a cross-sectional self-report questionnaire. In total, 2,691 questionnaires were returned; after excluding incomplete or invalid responses, 2,432 were retained for analysis (valid-response rate 90.38%). The final sample (*N* = 2,432) had a mean age of 20.3 years (SD = 1.6, range 17–26). By gender, 1,318 participants identified as women (54.2%), 1,092 as men (44.9%), and 22 selected other/prefer not to say (0.9%). Participants were distributed across academic years as follows: first-year 632 (26.0%), second-year 702 (28.9%), third-year 646 (26.6%), and fourth-year 452 (18.6%). Fields of study were grouped as business/economics 983 (40.4%), engineering/technology 902 (37.1%), humanities/social sciences 436 (17.9%), and other 111 (4.6%). Living arrangement was on-campus for 1,685 (69.3%) and off-campus for 747 (30.7%). By university, 980 (40.3%) were from Guangdong University of Finance and Economics, 796 (32.7%) from Jinan University, and 656 (27.0%) from Guangdong University of Technology. Participation was voluntary and anonymous; informed consent was obtained from all respondents prior to the survey.

### 3.2 Research tools

#### 3.2.1 Measurement of vigorous physical activity

Physical activity was assessed with the International Physical Activity Questionnaire–Short Form (IPAQ-SF), a seven-item self-report measure that records the frequency (days/week) and duration (minutes/day) of vigorous and moderate activities and walking performed during the last 7 days, plus weekday sitting time (Craig et al., 2003). Following the official scoring protocol (November 2005), minutes at each intensity are multiplied by standard MET values (walking = 3.3 METs; moderate = 4.0 METs; vigorous = 8.0 METs) and summed to yield total MET-minutes/week; data can also be classified into low, moderate, and high activity categories using established frequency–duration rules (including thresholds around 600 and 3,000 MET-min/week) (International Physical Activity Questionnaire (IPAQ) Research Committee, 2005). To reduce over-reporting, bouts <10 min are excluded and daily durations are truncated at 180 min per intensity (IPAQ Research Committee, 2005). The IPAQ-SF shows good test–retest reliability (Spearman *ρ* ≈ 0.8) and acceptable criterion validity versus accelerometry (median *ρ* ≈ 0.30) across 12 countries (Craig et al., 2003), with convergent evidence from a systematic review (Lee et al., 2011) and additional accelerometer validation (Dai, 2015); the Chinese version has demonstrated reliability and validity in Chinese-speaking populations (Macfarlane et al., 2007).

Operationalization of VPA for SEM. In the present study, VPA was operationalized as vigorous-intensity energy expenditure in MET-min/week, computed according to the IPAQ-SF scoring protocol as: vigorous days/week × vigorous minutes/day × 8.0 MET (with standard IPAQ data-cleaning rules applied, e.g., excluding bouts <10 min and truncating extreme daily durations). This continuous vigorous MET-min/week variable was used as the observed exogenous predictor in the SEM. Although the IPAQ-SF also provides moderate-intensity PA, walking, and total PA indices, these were not included in the primary SEM, as the *a priori* hypotheses focused specifically on vigorous activity.

#### 3.2.2 Measurement of mental well-being (WHO-5)

The WHO-5 Well-Being Index is a brief, widely used indicator of subjective psychological well-being (Topp et al., 2015). It comprises five positively worded items capturing core facets of positive mental well-being—feeling cheerful and in good spirits, calm/relaxed, energetic and vigorous, refreshed on waking, and engaged or interested in daily life—referenced to the past 2 weeks. Each item is rated on a six-point scale from 0 (at no time) to 5 (all of the time), yielding a total score from 0 to 25, with higher values indicating better well-being. In Chinese samples, the instrument shows good internal consistency (Cronbach’s *α* = 0.85) (Fung et al., 2022). A raw score below 13 is commonly used to flag poor well-being and to screen for possible depression.

For conceptual clarity, we treat the WHO-5 as an indicator of positive mental well-being (i.e., higher scores reflect better well-being). It is not a symptom checklist; when discussing depression/anxiety in the background literature, we distinguish mental-ill-health symptoms from positive well-being.

#### 3.2.3 Measurement of resilience

Psychological resilience was measured with the Connor–Davidson Resilience Scale–10 (CD-RISC-10), a validated short form comprising 10 items scored from 0 (not true at all) to 4 (true nearly all the time), summed to a 0–40 total (higher = greater resilience). The CD-RISC-10 shows a unidimensional structure and good reliability in undergraduate samples, and the Chinese version has been translated and psychometrically supported in Chinese populations, making it appropriate for use with Chinese university students (Wang et al., 2010).

#### 3.2.4 Measurement of physical self-esteem

Physical self-esteem was assessed via the Physical Self-Perception Profile (PSPP) (Fox and Corbin, 1989), focusing on the Physical Self-Worth (PSW) subscale as an index of global esteem in the physical domain. The PSPP evaluates perceived sport competence, physical condition, body attractiveness, physical strength, and physical self-worth using a structured alternative response format. The PSPP comprises five 6-item subscales, and the present study employed the 6-item PSW subscale. Items are administered in a structured alternative response format (paired contrasting statements) and scored on a 4-point scale (1–4); higher PSW scores indicate higher physical self-esteem. (Chen et al., 2025).

### 3.3 Methods of analysis

All analyses were conducted on the valid sample (*N* = 2,432). We first performed data screening (range checks, outliers, and <5% missingness), computed IPAQ-SF weekly minutes and MET-minutes, and created scale scores for WHO-5, CD-RISC-10, and the Physical Self-Worth (PSPP) subscale. In the SEM, VPA (vigorous MET-min/week) was entered as an observed predictor. Descriptive statistics and Pearson correlations were obtained, and internal consistency was evaluated with Cronbach’s *α* and McDonald’s *ω*. A confirmatory factor analysis (CFA) was then used to verify the measurement model for the three psychological constructs (resilience, physical self-esteem, mental well-being), with model fit judged by CFI/TLI ≥ 0.90, RMSEA ≤ 0.08, and SRMR ≤ 0.08. Clarification of software and analytical approach for final analyses. Descriptive statistics and reliability analyses (Cronbach’s *α*) were conducted in IBM SPSS. The final hypothesis tests were conducted using structural equation modeling (SEM) in R (lavaan package). All focal paths were estimated simultaneously, and model fit was evaluated using standard indices. Missing data in the SEM analyses were handled using full-information maximum likelihood (FIML) estimation, such that all available data contributed to parameter estimation under the missing-at-random assumption.

## 4 Result

### 4.1 Common method bias test

We used a single diagnostic—the Harman’s single-factor test. All measurement items (VPA from IPAQ-SF, CD-RISC-10, PSPP–Physical Self-Worth, and WHO-5) were entered into an unrotated exploratory factor analysis (principal axis factoring, eigenvalue >1 rule). The analysis extracted four factors with eigenvalues >1, and the first factor accounted for 29.6% of the total variance, which is below the commonly used 40% threshold. These results suggest that common method bias is unlikely to be a serious threat to the validity of the findings.

And the internal consistency of the multi-item psychological measures was evaluated using Cronbach’s *α* and McDonald’s *ω* (illustrative values for formatting only): WHO-5 (*α* = 0.87, *ω* = 0.88), CD-RISC-10 (*α* = 0.89, *ω* = 0.90), and PSPP-Physical Self-Worth (*α* = 0.91, *ω* = 0.92). Internal consistency indices do not apply to the IPAQ-SF VPA score because it is derived from frequency–duration computations rather than a homogeneous item scale.

### 4.2 Correlation analyses

We computed two-tailed Pearson correlations among vigorous physical activity (IPAQ-SF; MET-min/week), mental well-being (WHO-5), resilience (CD-RISC-10), and physical self-esteem (PSPP–Physical Self-Worth) for the valid sample (*N* = 2,432). Descriptive statistics are reported in Table 1 (VPA: range = 0–10,080, M = 1814.3, SD = 2106.7; mental well-being: range = 0–25, M = 14.9, SD = 4.3; resilience: range = 2–40, M = 25.6, SD = 5.1; physical self-esteem: range = 6–24, M = 16.0, SD = 2.9). As shown in Table 1, vigorous physical activity was positively correlated with mental well-being (*r* = 0.22, *p* < 0.01), resilience (*r* = 0.24, *p* < 0.01), and physical self-esteem (*r* = 0.28, *p* < 0.01). Both proposed mediators were also positively associated with mental well-being—resilience (*r* = 0.46, *p* < 0.01) and physical self-esteem (*r* = 0.39, *p* < 0.01)—and were moderately related to each other (*r* = 0.42, *p* < 0.01). Following Cohen’s guidelines, these effects were small-to-moderate in magnitude and aligned with the hypothesized positive direction. No correlation exceeded 0.50, suggesting multicollinearity is unlikely to be a concern. Overall, this pattern supports proceeding with the planned serial mediation analysis.

**Table 1:** Correlation analysis of variables (*N* = 2,432).

Variable	Range	M	SD	1	2	3	4
1. Vigorous physical activity (MET-min/week)	0–10,080	1814.3	2106.7	–			
2. Mental health (WHO-5)	0–25	14.9	4.3	0.22**	–		
3. Resilience (CD-RISC-10)	2–40	25.6	5.1	0.24**	0.46**	–	
4. Physical self-esteem (PSPP-PSW)	6–24	16.0	2.9	0.28**	0.39**	0.42**	–

*p* < 0.05, **p < 0.01.

### 4.3 Analysis of chain mediation effects

#### 4.3.1 Model fit analysis

Model–data fit was assessed with a confirmatory factor analysis (CFA) for the three latent constructs (resilience, physical self-esteem, mental well-being), followed by a structural equation model (SEM) specifying the hypothesized serial mediation with VPA as an observed predictor. The CFA showed good fit, *χ*^2^(186) = 578.42, *χ*^2^/df = 3.11, CFI = 0.956, TLI = 0.948, RMSEA = 0.046 [90% CI (0.042, 0.050)], SRMR = 0.036, and all standardized loadings were significant (0.62–0.86). The structural model also fit well, *χ*^2^(188) = 612.77, CFI = 0.952, TLI = 0.944, RMSEA = 0.047 [90% CI (0.043, 0.051)], SRMR = 0.039, and it outperformed a constrained model without indirect paths [Δ*χ*^2^(2) = 47.9, *p* < 0.001]. Collectively, these indices meet or exceed conventional thresholds (CFI/TLI ≥ 0.90; RMSEA/SRMR ≤0.08), supporting the adequacy of the model for testing the proposed serial mediation effects.

#### 4.3.2 Structural equation modeling results analysis

**Figure 1:**
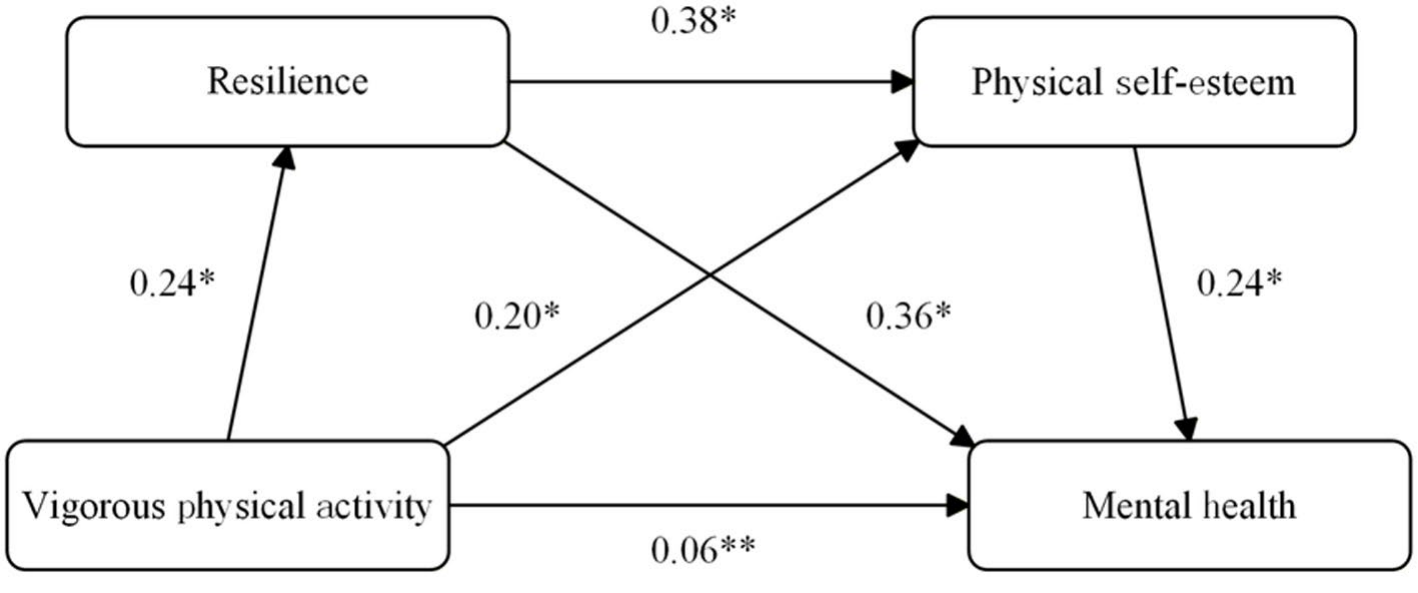
Structural equation model with standardized path coefficients (*β*). Significance levels are indicated as *p* < 0.05, *p* < 0.01, *p* < 0.001. No covariates were included in the SEM.

In the well-fitting SEM, vigorous physical activity (VPA) positively predicted resilience (*β* = 0.24, *p* < 0.001) and physical self-esteem (*β* = 0.20, *p* < 0.001), and resilience further predicted physical self-esteem (*β* = 0.38, *p* < 0.001). Both mediators were associated with better mental well-being (resilience → mental well-being: *β* = 0.36, *p* < 0.001; physical self-esteem → mental well-being: *β* = 0.24, *p* < 0.001). The direct path from VPA to mental well-being remained small but significant (*β* = 0.06, *p* < 0.01). Bias-corrected bootstrap tests (5,000 resamples) indicated significant indirect associations: via resilience [*β*_ind = 0.086, 95% CI (0.065, 0.110)], via physical self-esteem [*β*_ind = 0.048, 95% CI (0.034, 0.064)], and the serial pathway VPA → resilience → physical self-esteem → mental well-being [*β*_ind = 0.022, 95% CI (0.014, 0.031)]. The total indirect association was 0.156 (95% CI [0.129, 0.186]), yielding a total effect of VPA on mental well-being of 0.220. The model explained 5.8% of the variance in resilience, 22.1% in physical self-esteem, and 28.4% in mental well-being (see Figure 1).

#### 4.3.3 Analysis of mediating effects

Bias-corrected bootstrap tests (5,000 resamples; *N* = 2,432) showed that VPA was associated with mental well-being primarily via indirect associations consistent with the proposed mediators. The indirect association via resilience was significant [*β* = 0.086, 95% CI (0.065, 0.110)], as was the indirect association via physical self-esteem [*β* = 0.048, 95% CI (0.034, 0.064)]. The serial pathway VPA → resilience → physical self-esteem → mental well-being also reached significance [*β* = 0.022, 95% CI (0.014, 0.031)]. All confidence intervals excluded zero. Summed together, the indirect associations equaled 0.156, accounting for 70.9% of the total effect (*β*_total = 0.220; proportion = path effect/total effect). The direct association (*β* = 0.060) accounted for 27.3% of the total effect. The direct association of VPA on mental well-being remained small but significant [*β* = 0.060, 95% CI (0.015, 0.105)], indicating partial (complementary) mediation. Overall, these findings support H2 and H3 and corroborate the serial mediation hypothesis (H4) (see Table 2).

**Table 2:** Mediation effect path analysis.

Type of effect			95% Confidence interval		
Path	*β*	SE	LL	UL	Path effect proportion
Vigorous physical activity → mental health	0.060	0.023	0.015	0.105	27.3%
Vigorous physical activity → resilience → mental health	0.086	0.012	0.065	0.110	39.1%
Vigorous physical activity → physical self-esteem → mental health	0.048	0.008	0.034	0.064	21.8%
Vigorous physical activity → resilience → physical self-esteem → mental health	0.022	0.004	0.014	0.031	10.0%

## 5 Discussion

This study found that VPA is positively related to mental well-being in university students, with the association being statistically consistent with indirect pathways through resilience and physical self-esteem. In the structural model, VPA showed positive path coefficients with resilience (*β* = 0.24) and physical self-esteem (*β* = 0.20); resilience showed positive path coefficients with both physical self-esteem (*β* = 0.38) and mental well-being (*β* = 0.36), and physical self-esteem showed a positive path coefficient with mental well-being (*β* = 0.24). The direct association between VPA and mental well-being was small but significant (*β* = 0.06). Bootstrap analyses supported three significant indirect associations: via resilience [*β* = 0.086, 95% CI (0.065, 0.110)], via physical self-esteem [*β* = 0.048, 95% CI (0.034, 0.064)], and the serial route VPA → resilience → physical self-esteem → mental well-being [*β* = 0.022, 95% CI (0.014, 0.031)]. In aggregate, the indirect associations totaled 0.156, accounting for 70.9% of the total association (*β*_total = 0.220), which is consistent with complementary (partial) statistical mediation. Given the cross-sectional design, these pathways should be interpreted as model-implied statistical indirect associations consistent with the hypothesized ordering, rather than evidence of causal mechanisms or temporal precedence.

The pattern of results contributes to theory in two ways. First, it is consistent with stress-adaptation accounts in which engagement in higher-intensity activity may be linked to greater coping capacity and stress tolerance—captured here as resilience—which in turn is associated with better mental well-being. Second, it aligns with hierarchical models of the physical self that propose exercise-related experiences may relate to more positive body-related self-worth, which is in turn associated with well-being. The relative contributions—resilience-only (39.1%) exceeding physical self-esteem-only (21.8%) and the serial pathway (10.0%)—suggest that the indirect association via resilience accounted for a larger share of the total indirect association than the other components. However, the absolute magnitude of the indirect associations—particularly the serial pathway (*β* = 0.022)—was small, so conclusions about the strength of any specific mechanism should be made cautiously. The significant serial path is consistent with the possibility that resilience relates to more positive body-related appraisals, but this pathway appears modest in size and warrants replication in longitudinal/intervention studies.

These findings may have practical relevance for campus health promotion, while recognizing that intervention implications require prospective testing. Programs that increase opportunities for vigorous-intensity activity may be promising, and pairing activity with brief resilience-building components (e.g., goal setting, mastery reflection, stress-management drills) could be considered for evaluation in future trials. For example, in the Chinese university context, a mandatory PE class or campus fitness club could embed a 5–8 min ‘resilience micro-skill’ routine into each vigorous session: students set a weekly process goal, practice brief paced-breathing or cognitive reappraisal during high-intensity intervals, and complete a 2-min post-class mastery reflection (e.g., via a WeChat mini-program or learning platform) to connect effort with coping. Another feasible approach is to enhance physical self-esteem by designing sessions around competence and body functionality rather than appearance: instructors provide levelled exercise options and self-referenced feedback (e.g., progress tracked against one’s own baseline on fitness indicators used in the National Student Physical Health Standard), plus peer support and non-appearance-focused messaging that emphasizes strength, endurance, and health. The persistence of a small direct association between VPA and mental well-being further suggests that increasing access and adherence to vigorous activity may be associated with better mental well-being even when psychosocial correlates are not explicitly targeted, although causal conclusions cannot be drawn from the present design.

Several strengths bolster confidence in the results, including a large multi-university sample (*N* = 2,432), validated instruments (IPAQ-SF, CD-RISC-10, PSPP–Physical Self-Worth, WHO-5), and a well-fitting measurement and structural model with bias-corrected bootstrapping. Nonetheless, limitations warrant caution. The cross-sectional design precludes strong causal claims about temporal ordering among VPA, resilience, physical self-esteem, and mental well-being. All constructs were self-reported, so misclassification (e.g., activity over-reporting) and shared-method variance cannot be ruled out despite the Harman test suggesting no single-factor dominance. The sample comes from three universities in Guangdong Province, which may limit generalizability. Finally, the explained variance in the endogenous variables (*R*^2^ = 0.058 for resilience, 0.221 for physical self-esteem, and 0.284 for mental well-being) should be interpreted as small-to-moderate. In psychosocial research—where well-being and related constructs are shaped by multiple social, contextual, and individual determinants—such levels of explained variance are common and can still reflect meaningful effects, especially for a modifiable behavior like vigorous physical activity. Nevertheless, substantial variance remains unexplained, pointing to additional pathways (e.g., sleep, social connectedness, emotion regulation) that warrant investigation.

Future research should employ longitudinal or experimental designs with objective activity tracking to test temporal precedence and evaluate whether the proposed indirect pathways replicate under designs that support causal inference. Additional work could test measurement invariance and moderated mediation (e.g., by gender, year of study, baseline fitness). Micro-interventions that integrate brief resilience training and competence-focused feedback into vigorous activity sessions may help determine whether targeting both correlates yields additive benefits for student well-being.

In sum, VPA is linked to better mental well-being in university students chiefly through resilience and physical self-esteem, with a small but reliable direct association. Overall, the evidence is consistent with a serial pattern in which resilience is associated with stronger body-related self-worth, and these factors together help account for much of the VPA–mental well-being association, providing a mechanism-informed (but non-causal) rationale for future campus programs and intervention studies that combine vigorous activity with resilience-building and competence-enhancing components.

## 6 Conclusion

In conclusion, this multi-university study (*N* = 2,432) shows that vigorous physical activity is associated with better mental well-being in university students, with statistically significant but generally small indirect associations via resilience (*β* = 0.086), via physical self-esteem (*β* = 0.048), and serially through resilience → physical self-esteem (*β* = 0.022). Although the indirect components accounted for 70.9% of the total association, the total association was modest in size (*β*_total = 0.220), and the serial indirect component was particularly small; therefore, practical significance should be interpreted cautiously. Future longitudinal or experimental studies are needed to determine whether these indirect pathways replicate and whether they translate into meaningful intervention effects.

## 7 Limitation

Although Harman’s single-factor test did not indicate a dominant factor, the study relied entirely on self-report, single-source measures, which still implies potential common method variance and may inflate the observed associations among the focal constructs. In addition, self-reported physical activity is vulnerable to recall error and social desirability bias; therefore, vigorous physical activity levels may be overestimated. Future research could reduce these concerns by incorporating objective indicators of physical activity (e.g., accelerometers) and/or collecting key variables from multiple sources. Moreover, given the cross-sectional design and the absence of adjustment for potential confounders, the observed associations should be interpreted as correlational and may partly reflect unmeasured factors (e.g., age, sex, baseline health, socioeconomic status, or academic stress). Future longitudinal and/or intervention studies that incorporate key covariates and establish temporal precedence are needed to assess the robustness and causal plausibility of the proposed pathways.

## Data Availability

The raw data supporting the conclusions of this article will be made available by the authors, without undue reservation.
